# MicroRNA-182-5p Promotes Cell Invasion and Proliferation by Down Regulating *FOXF2*, *RECK and MTSS1* Genes in Human Prostate Cancer

**DOI:** 10.1371/journal.pone.0055502

**Published:** 2013-01-30

**Authors:** Hiroshi Hirata, Koji Ueno, Varahram Shahryari, Guoren Deng, Yuichiro Tanaka, Z. Laura Tabatabai, Yuji Hinoda, Rajvir Dahiya

**Affiliations:** 1 Department of Urology, San Francisco Veterans Affairs Medical Center and University of California San Francisco, San Francisco, California, United States of America; 2 Department of Pathology, San Francisco Veterans Affairs Medical Center and University of California San Francisco, San Francisco, California, United States of America; 3 Department of Oncology and Laboratory Medicine, Yamaguchi University Graduate School of Medicine, Yamaguchi, Japan; Children's Hospital Boston & Harvard Medical School, United States of America

## Abstract

Recently miR-182 has been reported to be over-expressed in prostate cancer (PC) tissues, however detailed functional analysis of miR-182-5p has not been carried out. The purpose of this study was to: 1. analyze the function of miR-182-5p in prostate cancer, 2. assess its usefulness as a tumor marker, 3. identify miR-182-5p target genes in PC, 4. investigate the potential for miR-182-5p inhibitor to be used in PC treatment. Initially we found that miR-182-5p expression was significantly higher in prostate cancer tissues and cell lines compared to normal prostate tissues and cells. Moreover high miR-182-5p expression was associated with shorter overall survival in PC patients. To study the functional significance of miR-182-5p, we knocked down miR-182-5p with miR-182-5p inhibitor. After miR-182-5p knock-down, prostate cancer cell proliferation, migration and invasion were decreased. We identified *FOXF2*, *RECK* and *MTSS1* as potential target genes of miR-182-5p using several algorithms which was confirmed by 3’UTR luciferase assay and Western analysis. Knock-down of miR-182-5p also significantly decreased *in vivo* prostate tumor growth. In conclusion this is the first report documenting that over-expression of miR-182-5p is associated with prostate cancer progression and potentially useful as a prognostic biomarker. Also knock down of miR-182-5p in order to increase expression of tumor suppressor genes *FOXF2*, *RECK* and *MTSS1* may be of therapeutic benefit in prostate cancer treatment.

## Introduction

Prostate cancer (PC) is one of the most common malignancies in U.S. males [1]. The etiology of PC is largely unknown, although several risk factors such as ethnicity, family history, and age are associated with the disease [2]. In addition, several dietary constituents have been linked to PC risk and prevention [3], [4]. As prostate-specific antigen (PSA) screening has spread, the number of curable patients has tended to increase. However, a significant number of patients with lymph node metastasis are identified during radical prostatectomy, making treatment more difficult [5].

Recently a number of miRNAs have been identified and reported to be important in several cancers [6]. MicroRNAs (miRNAs) are small non-coding RNAs approximately 22 nucleotides in length that are capable of regulating gene expression at both the transcription and translation levels [7]. MiRNAs bind to the 3’ untranslated region (UTR) of target mRNA and represses translation from mRNA to protein or induce mRNA cleavage, thereby regulating the expression of target genes such as tumor suppressor or oncogenes [8], [9]. Recently miR-182 has been reported to be over-expressed in prostate cancer [10]. However functional analysis of miR-182-5p has not been carried out in prostate cancer. Therefore we hypothesize that miR-182-5p may function as an oncogene and be a new molecular biomarker in prostate cancer. To test this hypothesis, we initially found that miR-182-5p expression was significantly higher in prostate cancer tissues compared with normal prostate tissues. Additionally the expression of miR-182-5p was significantly higher in prostate cancer cell lines (LNCaP, PC-3, DU145) compared with normal prostate epithelial cells (RWPE-1). For functional analysis studies, miR-182-5p was knocked down using a miR-182-5p inhibitor. We also used several algorithms to search for potential tumor suppressor genes as targeting for miR-182-5p. 3’UTR luciferase assay and Western analyses were performed to confirm direct interaction between miR-182-5p and these target genes. Finally we established stable low miR-182-5p expressing cell lines and performed *in vivo* studies in order to observe potential tumor suppression effects in a xenograft nude mouse model.

## Results

### miRNA-182 expression is significantly increased in prostate cancer tissues and correlated with overall survival

**Figure 1:**
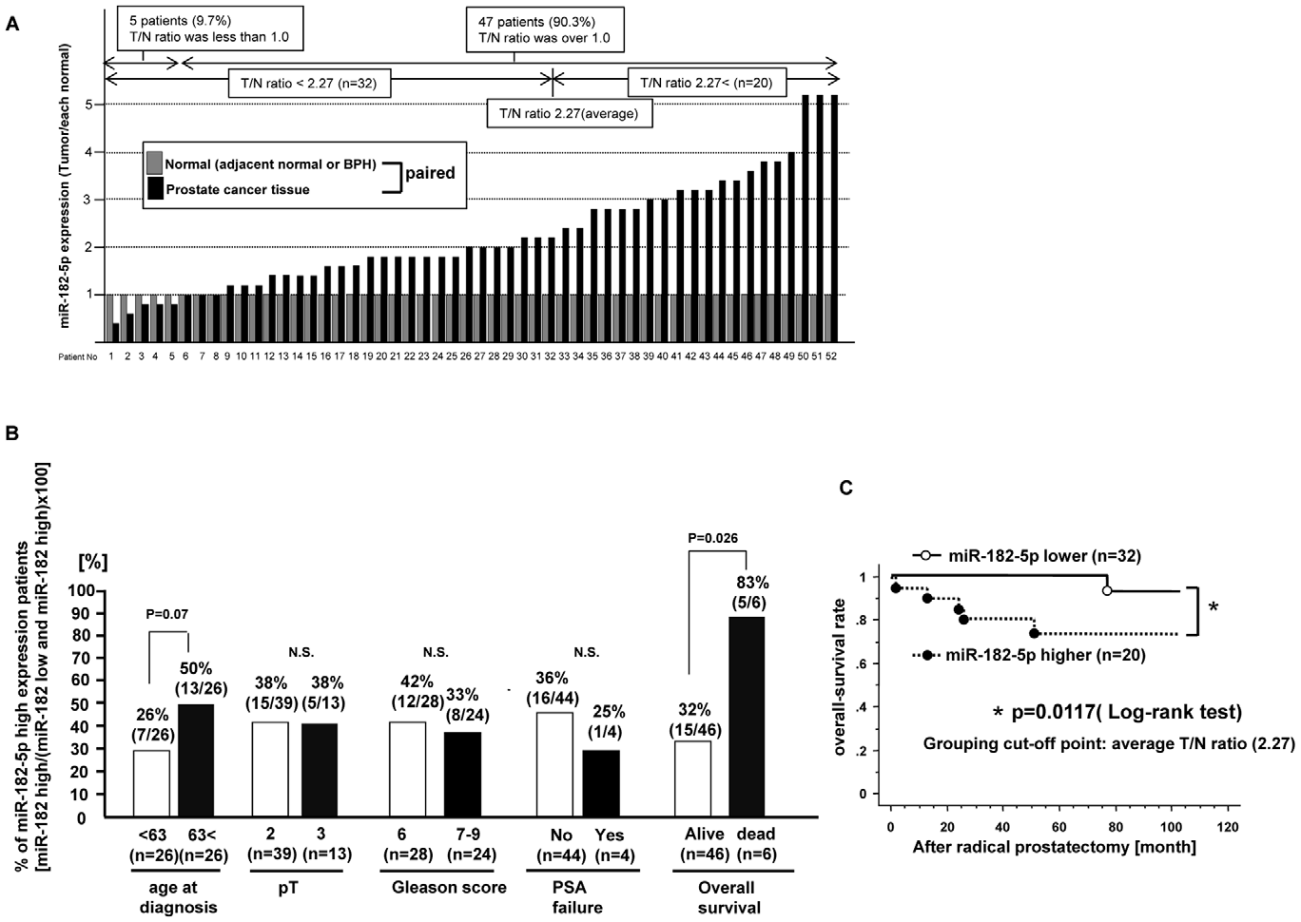
miR-182-5p expression in human prostate cancer tissues and normal prostate tissues and association with clinic-pathological parameters (q-PCR). A. Relative miR-182-5p expression [each tumor tissue (T)/normal prostate tissue (N)] based on real time PCR results, B. Association between miR-182-5p expression and clinic-pathological parameters (age at diagnosis, pT, Gleason score, PSA failure and overall survival) in prostate cancer tissues based on q-PCR results. C. Kaplan-Meier plots of over-all survival after radical prostatectomy.

MiR-182-5p expression levels in clinical samples (52 samples) were confirmed by real-time PCR. MiR-182-5p expression is shown as the ratio of tumor (T)/and normal (N) expression (T/N ratio) in each paired sample (**Figure 1**). Thus if the T/N ratio is over 1.0, miR-182-5p expression was judged to be higher in prostate cancer tissues compared to that in matched adjacent normal tissue. As shown in **Figure 1** **-A**, in 5 patients (9.7%), the T/N ratio was less than 1.0 while in the other 47 patients (90.3%), the T/N ratio was more than 1.0. Therefore miR-182-5p expression was significantly higher in prostate cancer tissues compared to matched normal prostate tissues. We divided the 52 prostate cancer patients into two categories based on the average T/N ratio (2.27) as follows: 1) high miR-182-5p expressing group (miR-182-5p T/N ratio higher than 2.27), 2) low miR-182-5p expressing group (miR-182-5p T/N ratio lower than 2.27). We then investigated the association of miR-182-5p and several clinical parameters as shown in **Figure 1-B**. Kaplan Meier plots showed that overall survival was significantly shorter in the high miR-182-5p expressing group (p value = 0.0117, Log-rank test) **(** **Figure 1-C** **)**.

### miRNA-182-5p expression is significantly increased in prostate cancer cell lines

**Figure 2:**
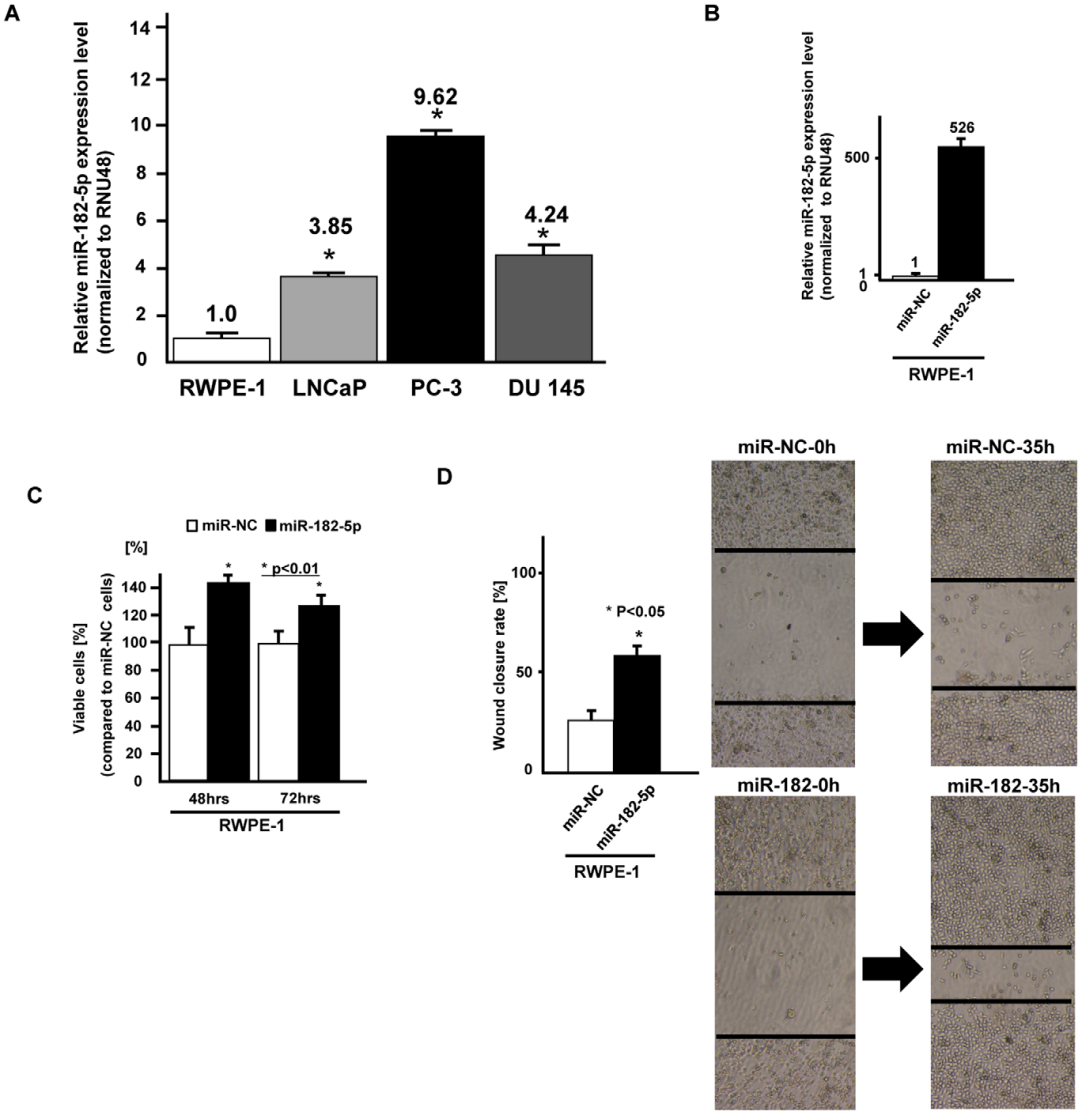
Expression of miR-182-5p in cell lines and effect of miR-182-5p overexpression on normal prostate cells (RWPE-1). A. The expression of miR-182-5p was significantly higher in three prostate cancer cell lines compared to a normal prostate cell line (RWPE-1), B. Relative miR-182-5p expression (miR-NC or miR-182-5p precursor transfected RWPE-1 cells), B. cell viability assay (miR-NC or miR-182-5p precursor transfected RWPE-1 cells), C. Wound healing assay (miR-NC or miR-182-5p precursor transfected RWPE-1 cells). Error bars represent ±S.D. (standard deviation).

MiR-182-5p expression was significantly higher in prostate cancer cell lines (LNCaP, PC-3 and DU145) compared to the normal prostate cell line (RWPE-1) (**Figure 2A**).

### Effect of microRNA-182-5p over-expression on cell viability and migration in a normal prostate cell line

To confirm the function of miR-182-5p, we transfected miR-182-5p precursor into a normal prostate cell line (RWPE-1). At 48 hours after transfection of miR-NC or miR-182-5p precursor into RWPE-1 cells, the miR-182-5p expression level was verified by real time PCR (fold change; 526, **Fig. 2-B**). Then cell viability (MTS assay) and wound healing assays were performed using RWPE-1 cells transiently transfected with miRNA-182-5p precursor. We observed significantly increased cell proliferation (**Fig. 2-C**) and migration (**Fig. 2-D**) in miRNA-182-5p transfected cells compared to miR-NC transfected cells.

### Effect of microRNA-182-5p knock down on cell viability, invasion and migration in PC cell lines

**Figure 3:**
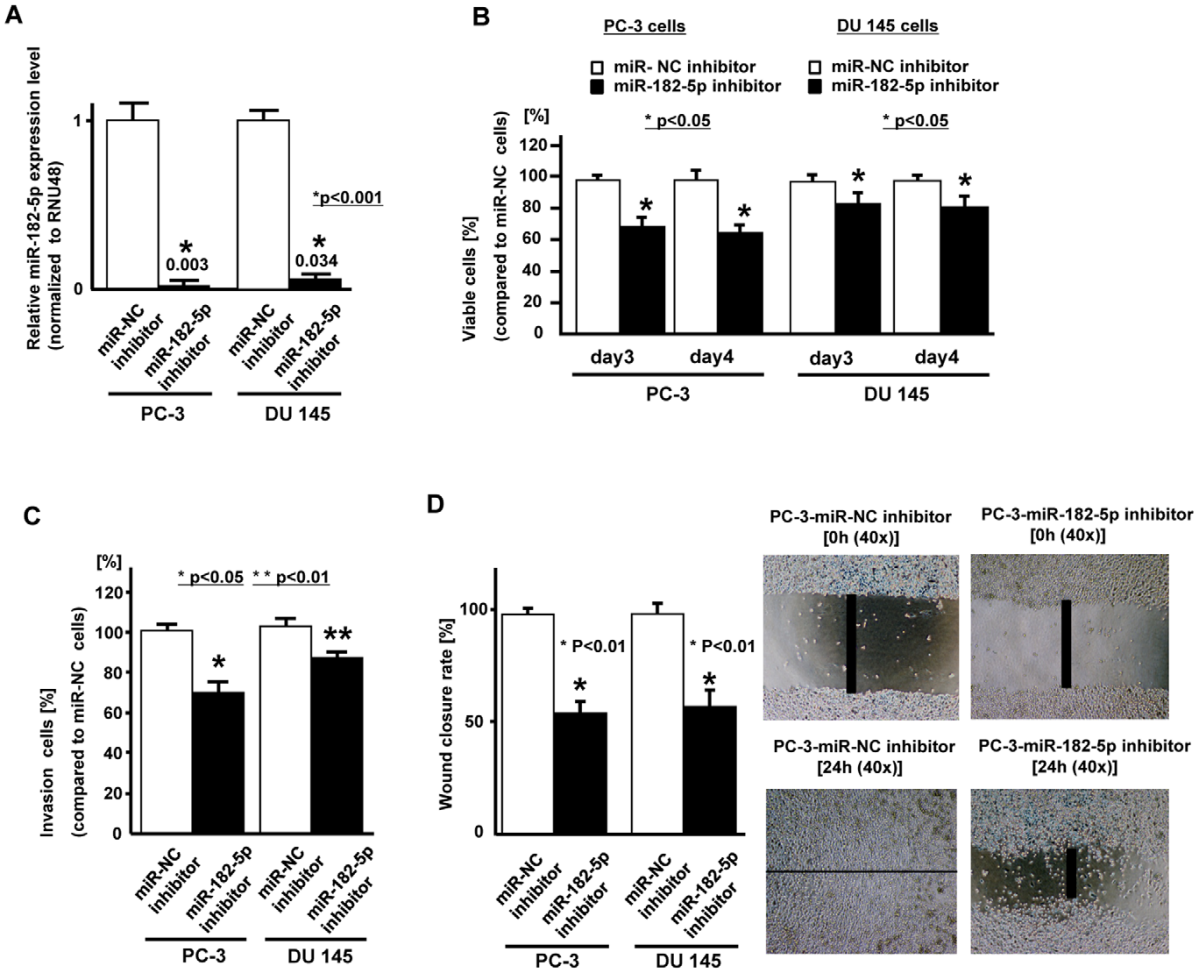
Effect of miR-182-5p knock down on prostate cancer cells (PC-3, DU145). Two prostate cancer cell lines (PC-3 and DU145) were transiently transfected with either miR-182-5p inhibitor or miR-negative control (miR-NC-inhibitor). A. Relative miR-182-5p expression (miR-NC inhibitor or miR-182-5p inhibitor transfected PC cells), B. Cell viability assay (miR-NC inhibitor or miR-182-5p inhibitor transfected PC cells), C. Invasion assay, D. Wound healing assay (24 hours). Error bars represent±S.D. (standard deviation).

We used two prostate cancer cell lines (PC-3 and DU145) with high miR-182-5p expression for further experiments since the miR-182-5p expression was significantly higher in these cell lines. At 48 hours after transfection of inhibitor-NC or miR-182-5p inhibitor into PC-3 and DU145 cells, the miR-182-5p knock-down was verified by real time RT-PCR (0.003, 0.034, respectively) (**Fig. 3-A**). Cell viability (MTS assay), migration and invasion assays were performed (**Fig. 3-B, 3-C, 3-D**) and we observed a significant decrease in cell proliferation (**Fig. 3-B**), invasion (**Fig. 3-C**) and migration (**Fig. 3-D**) in miRNA-182-5p knockdown PC-3 and DU-145 cells compared to inhibitor-NC transfected cells.

### miR-182-5p directly regulates FOXF2, RECK and MTSS1

We identified three tumor suppressor genes (*FOXF2*, *RECK, MTSS1*) as potential target genes for miR-182-5p based on three algorithms (miRDB, TargetScan, microRNA.org).

**Figure 4:**
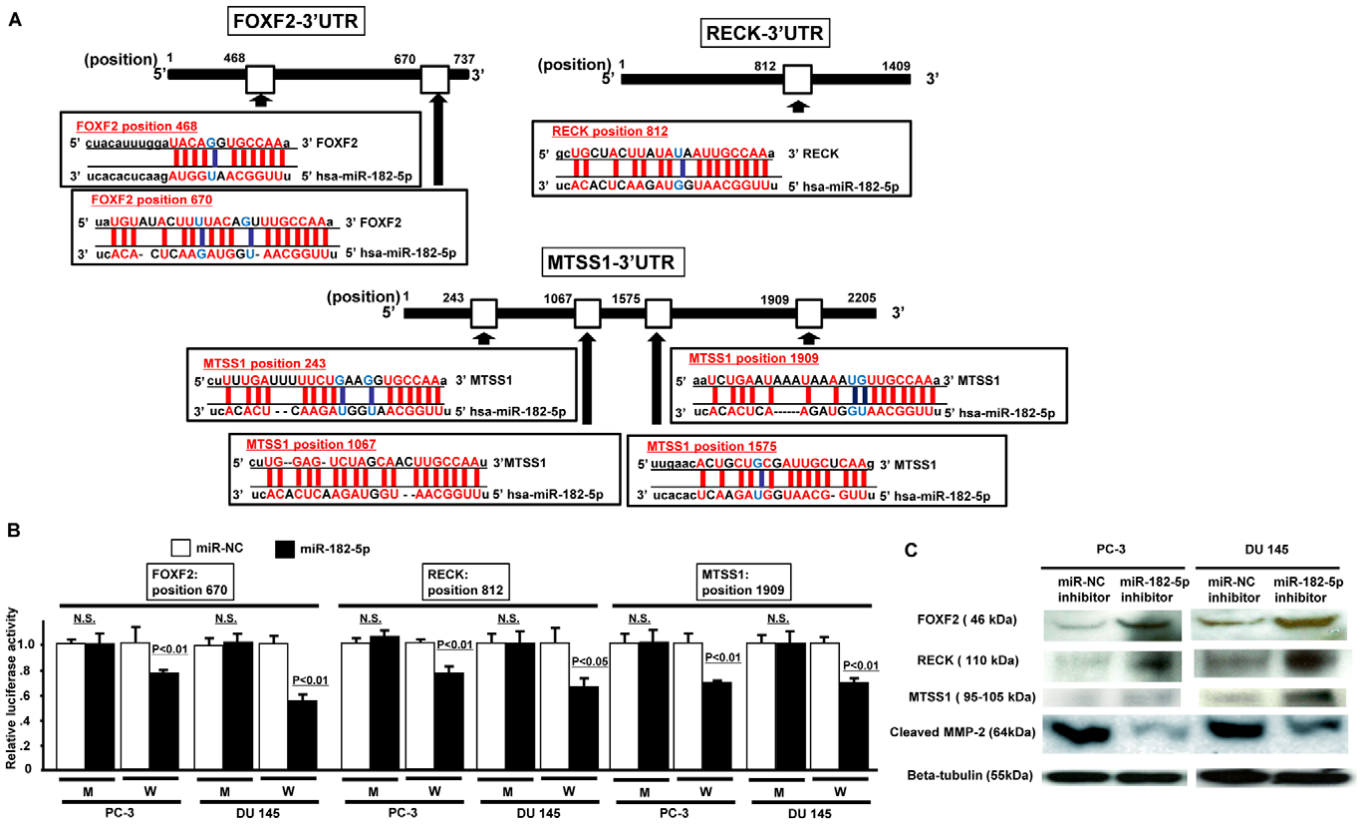
miR-182-5p binds to the 3’ UTR of FOXF2, RECK and MTSS1 mRNAs and down-regulates expression. A. FOXF2, RECK and MTSS1 3’UTR position and complementary miR-182-5p sequence. B. 3’UTR Luciferase assay (miR-NC and miR-182-5p precursor), Error bars represent±S.D. (standard deviation). C. Protein expression of FOXF2, RECK, MTSS1, MMP-2 and beta-tubulin in miR-NC inhibitor or miR-182-5p inhibitor transfected prostate cancer cells (PC-3, DU145).

FOXF2 mRNA has two potential binding sites for miR-182-5p within its 3’ UTR (**Fig. 4-A**). The relative luciferase activity is shown as a ratio of firefly and renilla luciferase activity for each sample. Among the two sites, the relative luciferase activity was significantly decreased at position 670 in miR-182-5p precursor transfected cells (**Fig. 4-B**). RECK mRNA has one potential binding site (position 812) for miR-182-5p within its 3’ UTR (**Fig. 4-A**). The relative luciferase activity was significantly decreased when the position 812 construct was used in miR-182-5p precursor transfected cells (**Fig. 4-B**). MTSS1 mRNA has four potential binding sites for miR-182-5p within its 3’UTR and the relative luciferase activity was significantly decreased at position 1909 in miR-182-5p precursor transfected cells (**Fig. 4-B**). With mutated plasmids (shown as “M”), there was no significant difference in luciferase activity between controls and miR-182-5p precursor transfectants (**Fig. 4-B**). Since target gene protein expression is low in PC-3 and DU145, we performed Western analysis to observe any changes in protein expression with miR-182-5p inhibitor. As shown in Figure 4-C, protein expression of target genes was significantly increased after miR-182-5p inhibitor transfection (**Fig. 4-C**). This result suggests that the FOXF2, RECK and MTSS1 mRNAs are direct targets of miR-182-5p.

We also confirmed active MMP-2 protein expression since it is a downstream effector of RECK. As shown in **Figure 4-C**, cleaved MMP-2 (active type) protein expression was significantly decreased in miR-182-5p inhibitor transfected cells.

### miR-182-5p inhibitor effect on tumor growth in an in vivo nude mouse model

**Figure 5:**
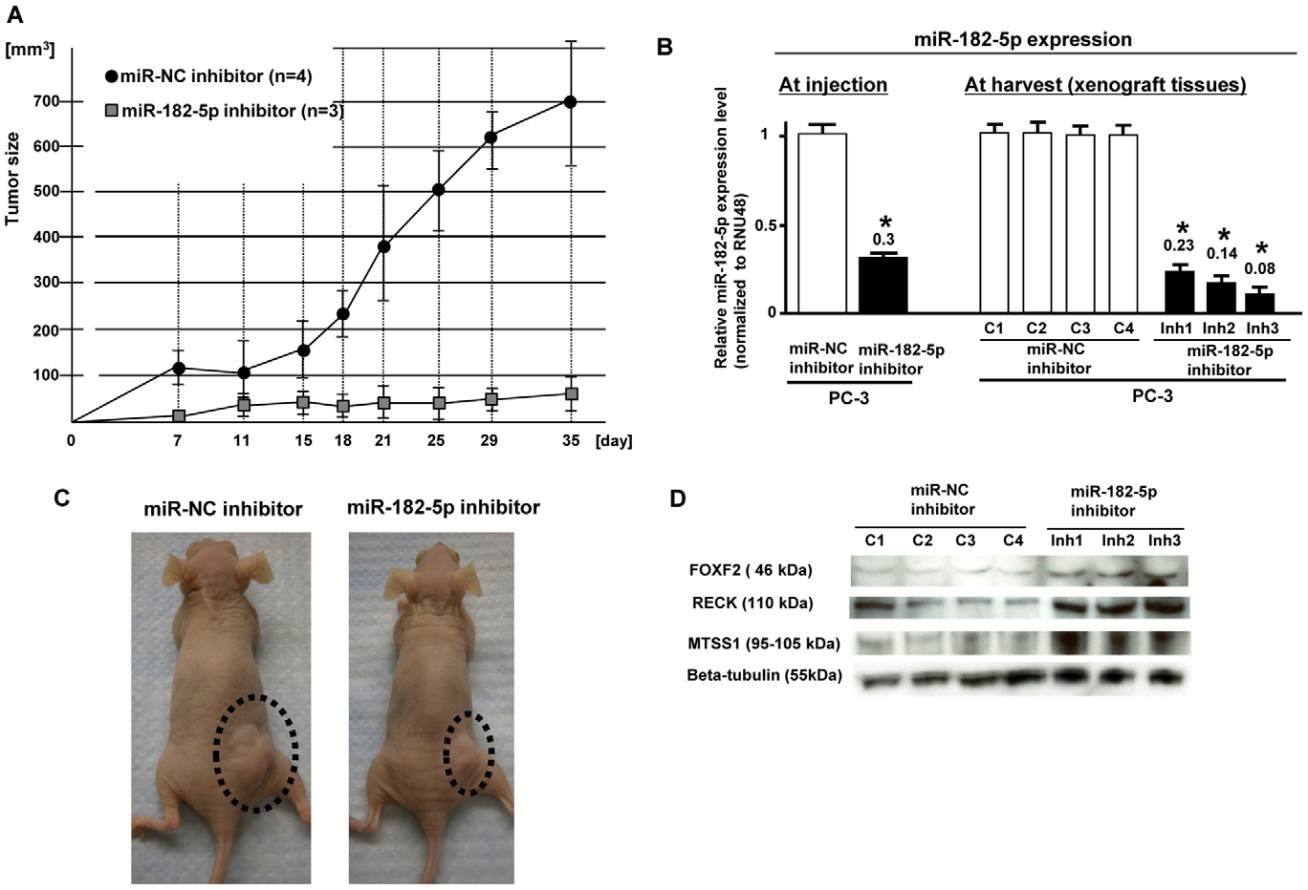
Inhibition of *in vivo* tumor growth by miR-182-5p knockdown in PC3 cells. A. Time record of tumor size in miR-NC and miR-182-5p inhibitor xenografts. B. Relative miR-182-5p expression at injection of cells and harvest of xenografts. Error bars represent ±S.D. (standard deviation). MiR-182-5p expression was significantly lower in the miR-182-5p inhibitor group. C. Typical gross appearance of nude mouse tumors in control and miR-182-5p inhibitor groups. D. Expression of FOXF2, RECK and MTSS1 protein in tumor xenografts.

In order to observe miR-182-5p inhibitor effects on tumor growth in nude mice, we used a lentiviral based system and established stable low miR-182-5p expressing PC-3 cells and controls (**Fig. 5-A**). We used 8 mice (4 mice; stable low miR-182-5p, 4 mice; control) and observed that stable low miR-182-5p expressing cell lines had significantly reduced tumor volume in nude mice compared to controls (**Fig. 5-B**). Since we could not detect tumor in one of the low miR-182-5p expression groups, it was excluded from the experiment. We measured microRNA levels in xenograft tumors (miR-182-5p-inhibitor and inhibitor-NC), and found that miR-182-5p expression was significantly lower in miR-182 inhibitor xenografts compared to that of inhibitor-NC xenografts (**Fig. 5-B**). Typical gross appearance pictures of tumors are shown in **Fig. 5-C**. As shown in **Figure 5-D**, protein expression of the three target genes (FOXF2, RECK, MTSS1) was significantly higher in stable low miR-182-5p expressing xenograft tumor tissues.

### Effect of over-expression of RECK and MTSS1 on prostate cancer cells (PC-3 and DU 145) function

**Figure 6:**
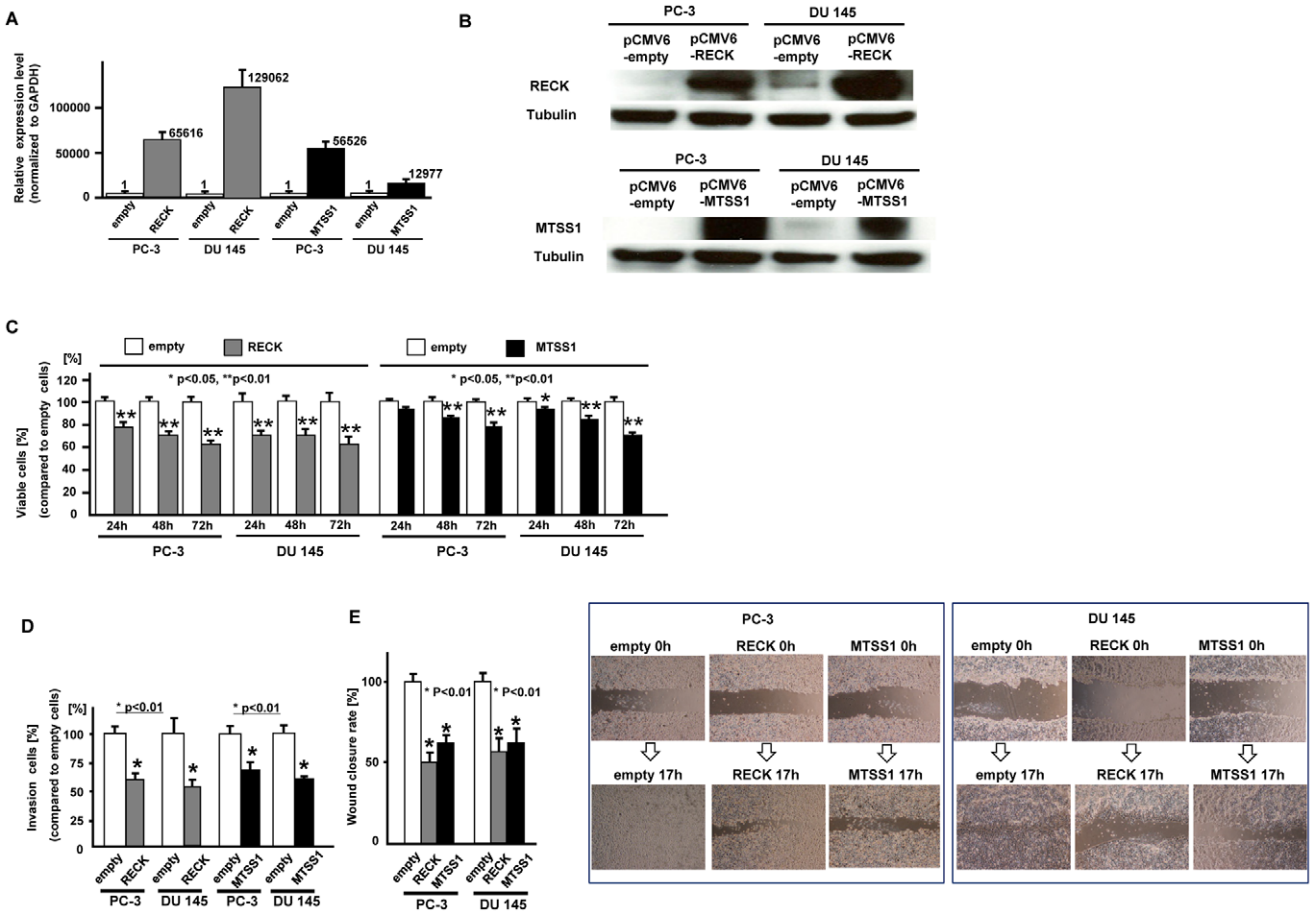
Effect of RECK and MTSS1 over-expression on prostate cancer cell function. At 24 hours after transfection of either pCMV6-empty, pCMV6-RECK or pCMV6-MTSS1 into prostate cancer cells (PC-3 and DU 145), RECK and MTSS1 expression levels were verified by real time RT-PCR (A) and Western analysis (B) C. Cell viability assay, D. Invasion assay, E. Wound healing assay, Error bars represent±S.D. (standard deviation).

To examine the function of RECK and MTSS1, we overexpressed RECK and MTSS1 in prostate cancer cells which were confirmed by measuring mRNA (**Fig. 6-A**) and protein expression levels (**Fig. 6-B**). Then we performed several functional analyses. As shown in Figure 6, cell viability (**Fig. 6-C**), invasion (**Fig. 6-D**) and migration (**Fig. 6-E**) were significantly inhibited in RECK and MTSS1 transfected prostate cancer cells.

### Effect of FOXF2 siRNA knockdown on cell proliferation, invasion and migration in prostate cancer cells

**Figure 7:**
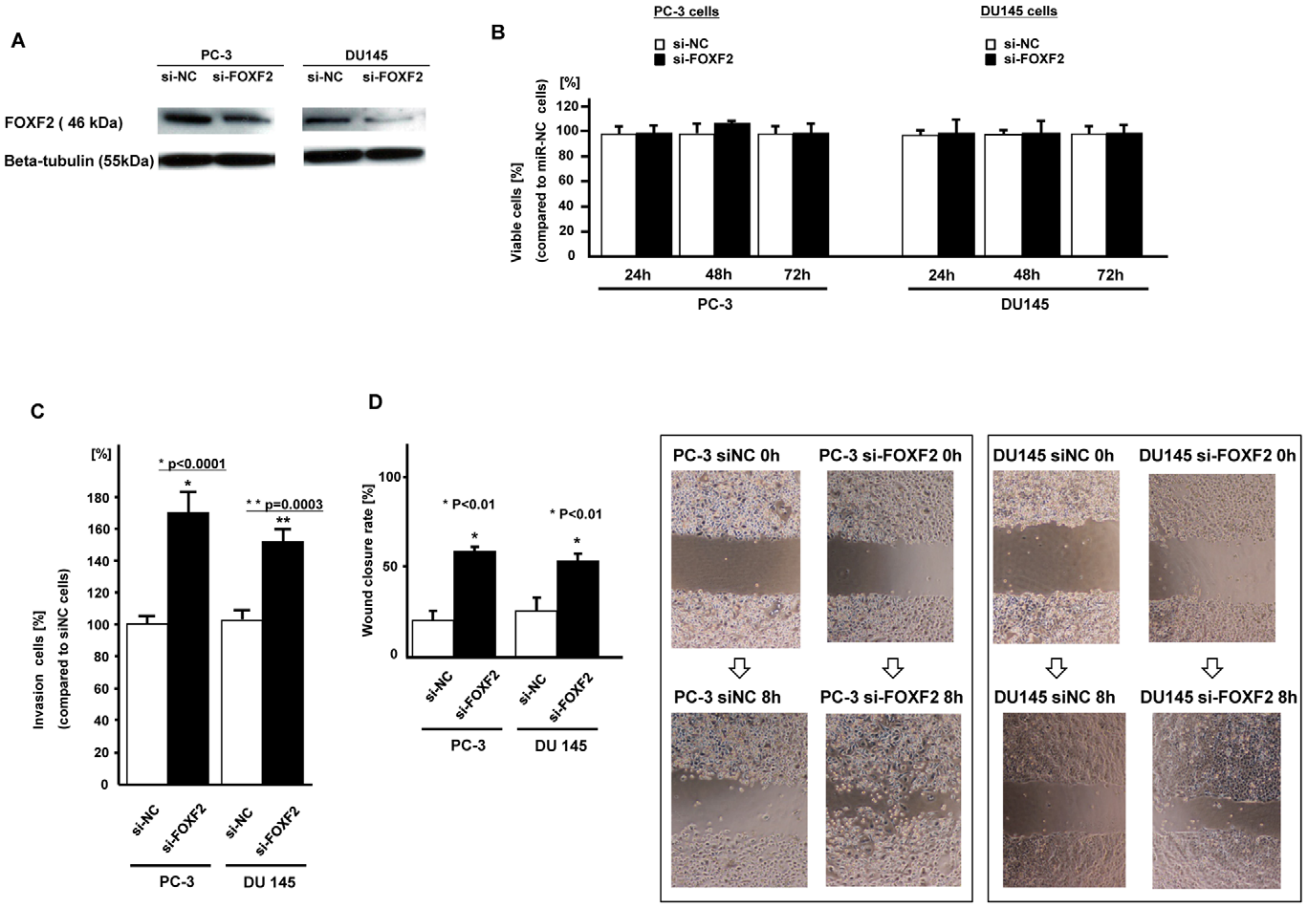
Effect of FOXF2 knockdown on prostate cancer cells (PC-3, DU145). A. FOXF2 protein expression (si-NC or si-FOXF2 transfected PC cells). B. Cell viability assay (si-NC or si-FOXF2 transfected PC cells). C. Invasion assay, D. Wound healing assay (8 hours), Error bars represent±S.D. (standard deviation).

After FOXF2 siRNA knock down (**Fig. 7-A**), there was no difference in cell proliferation between si-NC and si-FOXF2 transfected PC cells (**Fig. 7-B**). However cell invasion and wound healing were significantly increased in si-FOXF2 transfected PC cells (**Fig. 7-C**, **Fig. 7-D**).

## Discussion

Several microRNAs have been identified as oncogenic in prostate cancer based on their increased expression in prostate cancer tissues or prostate cancer cell lines [10], [11]–[14]. Functional analyses have been performed with some of these oncogenic microRNAs. For example, miR-21 has been reported to promote invasion and apoptosis resistance in prostate cancer cells [15], [16] and miR-125b promoted growth of prostate cancer xenograft tumors by targeting pro-apoptotic genes [14]. MiR-373 and miR-520c have also been reported to promote tumor invasion and metastasis in prostate cancer [12].

One report has shown that miR-182 is a tumor suppressor in a lung cancer cell line [17], while miR-182-5p has been reported to be an oncogene in several cancers [18]–[23]. Segura et al reported that aberrant miR-182-5p expression promoted melanoma metastasis [18]. Liu et al found that miR-182-5p overexpression increased tumour transformation and tumor invasiveness *in vitro* and enhanced metastasis *in vivo* in ovarian cancer cells [21]. Therefore only two reports have shown that miR-182-5p is an oncogene based on functional analysis in melanoma and ovarian cancer [18], [21].

Similar to our results, two other studies found miR-182-5p expression to be significantly higher in prostate cancer tissues compared to normal prostate tissues [10], [22], however they did not perform functional analysis. In our study, we observed that miR-182-5p expression was significantly higher in prostate cancer tissues and prostate cancer cell lines compared to normal prostate tissues and cell lines. Interestingly we found that high miR-182-5p expression was correlated with shorter overall survival after radical prostatectomy in prostate cancer patients. Thus our next aim was to elucidate whether miR-182-5p functions as an oncogene in prostate cancer. To address this question, we initially transfected miR-182-5p into a normal prostate cell line (RWPE-1) and observed that miR-182 increased cell proliferation and wound healing ability. We also performed *in vitro* functional analyses using a miR-182-5p inhibitor in prostate cancer cells. Since miR-182-5p expression was higher in all three prostate cancer cell lines (LNCaP, PC-3, DU145) compared to normal prostate epithelial cells, we used the two highest miR-182-5p expressing cell lines (PC-3 and DU145) for functional knockdown analyses. As expected, miR-182-5p knockdown decreased cell proliferation, migration and invasion in these cell lines. As it has previously been reported that miR-182-5p overexpression promoted melanoma metastasis and was oncogenic in nature, our results also indicate that miR-182-5p may be an oncogene in prostate cancer cell lines.

Our next aim was to identify target genes of miR-182-5p since microRNAs exert their effects by regulating expression of other target genes. We used several algorithms (miRDB, microRNA.org, TargetScan) and identified three potential target genes (FOXF2, RECK, MTSS1). As shown with 3’UTR luciferase assay and Western analyses, the three potential target genes (FOXF2, RECK, MTSS1) expression was regulated by miR-182-5p.

Of the three target genes (RECK, MTSS1, FOXF2), RECK overexpression was reported to decrease cell invasion by inhibiting MMPs including MMP-2 [24]. RECK expression has been reported to be down regulated in several cancers including prostate cancer [24]. In our study, we observed that miR-182 inhibitor decreased active-MMP-2 expression in prostate cancer cell lines as well as up-regulation of RECK protein. Additionally we investigated RECK function by over expressing it in prostate cancer cell lines (PC-3 and DU 145). As shown, RECK inhibited cell migration and invasion in prostate cancer cells. Rabien et al showed that RECK overexpression decreased the invasive potential of DU 145 cells [25] and their results are similar to ours. However we observed that RECK inhibited cell proliferation in two prostate cancer cell lines (PC-3 and DU 145), while Rabien et al found no effect of RECK on cell proliferation in DU 145 cells. However RECK has been reported to decrease cell proliferation in other cancers [26], [27]. Thus additional experiments are required to clarify these differences. So far miR-21 and miR-222 have been found to target RECK in prostate cancer and gastric cancer [28], [29] but there have been no reports about miR-182-5p and RECK. However our results show that miR-182-5p directly regulates RECK expression in prostate cancer cell lines.

MTSS1 (metastasis tumor suppressor-1) was initially identified as a tumor suppressor gene in bladder cancer [30]. Recently the function of MTSS1 was examined in prostate cancer cell lines and found to significantly suppress cell migration and proliferation [31]. In addition miR-182 has been reported to down-regulate MTSS1 and promote metastasis of hepatocellular carcinoma [32], a result very similar to ours.

FOXF2 has been reported to down regulate MMP-1 and up-regulate TIMP3, a known inhibitor of MMPs [33]. Moreover FOXF2 decreases Wnt5a [34], which acts through non-canonical Wnt signaling pathways to promote angiogenesis and cell survival. Wnt5a expression in prostate cancer tissues has been correlated with high Gleason scores and biochemical prostate cancer relapse [35]. Knockdown and overexpression of Wnt5a in human prostate cancer cell lines reduced and stimulated respectively, invasion activity [35]. Furthermore Wnt5a induced the expression of metalloproteinase-1 (MMP-1) in prostate cancer [34], [35]. In our study, FOXF2 knock down increased cell invasion and migration in prostate cancer cell lines. Thus our results suggest that onco-miR-182-5p may be involved in the regulation of these invasion and metastatic suppressor genes. Recently miR-301 was defined as an oncogene and it was reported to mediate proliferation via regulating FOXF2 in breast cancer [36]. Except for miR-301, no studies have shown direct regulation of FOXF2 by a miRNA.

We also investigated the possible use of miR-182-5p inhibitor as a potential PC treatment. To accomplish this, we used an *in vivo* nude mouse xenograft model with stable low miR-182-5p expressing prostate cancer cells for this study. The tumor volume in nude mice was significantly decreased in low miR-182-5p expressing cells compared to control cells. Additionally the levels of FOXF2, RECK and MTSS1 were significantly higher in xenograft tissues from low miR-182-5p expressing prostate cancer cells. These results indicate that knockdown of miR-182-5p increased expression of these tumor suppressor target genes *in vitro* and *in vivo*. Since we focused on three target genes (*RECK*, *MTSS1* and *FOXF2*) and investigated only 52 clinical samples, additional studies will be needed to elucidate the role of miR-182-5p in prostate cancer and its use in clinical applications.

In conclusion this is the first report documenting that over-expression of miR-182-5p is associated with prostate cancer progression and potentially useful as a prognostic biomarker. Additionally knocking down of miR-182-5p with inhibitor may be of therapeutic benefit for enhancing expression of tumor suppressor genes FOXF2, RECK and MTSS1 in prostate cancer cells.

## Materials and Methods

### Ethics Statement

Formalin-fixed, paraffin-embedded (FFPE) prostate cancer samples were obtained from the San Francisco Veterans Affairs (VA) Medical Center. Written informed consent was obtained from all patients and the study was approved by the UCSF Committee on Human Research (Approval number: H9058-35751-01).

### Animals

All animal care was in accordance with the guidelines of the San Francisco Veterans Affairs Medical Center and the study was approved by the San Francisco VA IACUC (Protocol number: 08-003-01). Animal users have completed training programs to handle and work with mice through AALAS (American Association for Laboratory Animal Science) prior to animal experiments. A total of 8 nude mice (strain BALB/c nu/nu; Charles River Laboratories, Inc., Wilmington, MA) were used and initially prostate cancer cells were subcutaneously injected and tumor size was monitored as mentioned on page 7. After tumor growth, mice were euthanized with an overdose of isoflurane by inhalation. Then xenograft tissue was removed.

### Clinical samples

A total of 52 patients with pathologically confirmed prostate cancer were enrolled in this study (Veterans Affairs Medical Center at San Francisco). Samples were obtained from the patients after written informed consent. Detailed patient information is shown in **Table 1**.

**Table 1:** Prostate cancer patient information (n = 52).

age (yrs)	mean	63 yrs (49–83)
Preoperative PSA (ng/ml)	less than 10	n = 40
	10 or greater	n = 11
	unknown	n = 1
		
pathological stage	pT2	n = 40
	pT3	n = 12
Gleason score (LCM sample)*	6	n = 28
	7	n = 21
	8	n = 2
	9	n = 1
PSA failure	No	n = 44
	Yes	n = 4
	unknown	n = 4
Overall survival after operation	survive	n = 46
	death	n = 6

### Cell culture

Normal prostate epithelial cells (RWPE-1; ATCC number-CRL-11609) and prostate cancer cell lines (LNCaP; ATCC number-CRL-1740, PC-3; ATCC number-CRL-1435, DU145; ATCC number-HTB-81) were purchased from the American Type Culture Collection (Manassas, VA). The prostate cancer cell lines were cultured in RPMI 1640 medium supplemented with 10% heat-inactivated fetal bovine serum. RWPE-1 cells were cultured in keratinocyte-SFM (GIBCO/Invitrogen, Carlsbad, CA, USA). When purchased, permanent stocks of cells were prepared and all cells were stored at −80 degree until use. Cells were used for experiments within 6 months.

### Total RNA and protein extraction

RNA (microRNA and total RNA) was extracted from formalin-fixed, paraffin-embedded (FFPE) human prostate cancer (n = 52) and matched adjacent non-cancerous normal prostate tissues (n = 43) or benign prostate hyperplasia (BPH) (n = 9) using a miRNeasy FFPE kit (Qiagen) after laser capture micro-dissection based on a pathologist reviews. RNA (microRNA and total RNA) was also extracted from human cell lines using a miRNeasy mini kit (QIAGEN) and extracted from xenograft tissues homogenized in 1 ml TRIzol reagent (Invitrogen, Carlsbad, CA) then purified with RNeasy columns (QIAGEN). To digest DNA, a Qiagen RNase-Free DNase kit was used. Cells were lysed with RIPA buffer (Pierce, Brebieres, France) containing protease inhibitors (Sigma, St. Louis, MO). Protein was extracted from xenograft tissues using Tissue Protein Extraction Reagent (T-PER) (Thermo Scientific, Rockford, IL). Protein quantification was done using a BCA protein assay kit (Pierce, Brebieres, France).

### MicroRNA and microRNA inhibitor transfection

Anti-miR™ miRNA inhibitor [negative control (miR-NC inhibitor) or miR-182-5p inhibitor (miR-182-5p inhibitor), Ambion] were transiently transfected into cancer cells with siPORT NeoFX Transfection Agent (Ambion) according to the manufacturer’s instructions. Pre-miR™ miRNA precursors [negative control (miR-NC) or hsa-miR-182-5p (miR-182-5p), Ambion] were transfected into cells with Lipofectamine 2000 (Invitrogen) according to the manufacturer’s instructions.

### Cell viability, cell invasion and wound healing assay

Cell viability was measured 3 days after transfection (miR-NC inhibitor/miR-182-5p inhibitor transfectant) with MTS (CellTiter 96 Aqueous One Solution Cell Proliferation Assay, Promega). Data are the mean ± S.D. of 3 independent experiments. Cell invasion assays were performed with the CytoSelect 24-well cell invasion assay kit (Cell BioLab, San Diego, CA) according to the manufacturer’s instructions. Transfected cells (miR-NC inhibitor/miR-182-5p inhibitor transfectant-48 hours) were re-suspended in culture medium without FBS and placed in the upper chamber in triplicate. After 48 hours incubation at 37° C (5% CO2), cells migrating through the membrane were stained. The results were expressed as invaded cells quantified at OD 560 nm. Wound healing assay was performed with the CytoSelect 24-well wound healing assay kit according to the manufacturer’s instructions. To generate a wound field, transfected cells (miR-NC inhibitor or miR-182-5p inhibitor transfectant-48 hours transfection) were cultured until they formed a monolayer around the insert. After removing the insert, a 0.9 mm open wound field was generated and cells were allowed to migrate from either side of the gap. Wound closure was monitored and the percent closure was measured. [Percent closure rate (%)  =  migrated cell surface area/total surface area x100)].

### Quantitative real-time RT-PCR

Quantitative real-time RT-PCR was performed in triplicate with an Applied Biosystems Prism 7500 Fast Sequence Detection System using TaqMan universal PCR master mix according to the manufacture’s protocol (Applied Biosystems Inc., Foster City, CA, USA). The TaqMan probes and primers were purchased from Applied Biosystems. RNU48 was used as endogenous control. Levels of RNA expression were determined using the 7500 Fast System SDS software version 1.3.1 (Applied Biosystems).

### Western analysis

Total cell protein (15–20 µg) was used for Western blotting. Samples were resolved in 4–20% Precise Protein Gels (Pierce, Brebieres, France) and transferred to PVDF membranes (Amersham Biosciences, Fairfield, CT). The membranes were immersed in 0.3% skim milk in TBS containing 0.1% Tween 20 for 1 hour and probed with primary polyclonal and monoclonal antibody against FOXF2 (#ab23306, Abcam, Cambridge, MA), RECK (#3433, Cell Signaling Technology), MTSS1 (#4385, Cell Signaling Technology), MMP−2 (#4022, Cell Signaling Technology) and beta-tubulin (#2128, Cell signaling Technology) overnight at 4°C. Blots were washed in TBS containing 0.1% Tween20 and labeled with horseradish peroxidase (HRP)-conjugated secondary anti-mouse or anti-rabbit antibody (Cell signaling Technology, Beverly, MA). Proteins were enhanced by chemiluminescence (Amersham ECL plus Western Blotting detection system, Fairfield, CT) for visualization. The protein expression levels were expressed relative to beta-tubulin levels.

### Plasmid construction and 3’UTR-Luciferase assay

We constructed individual plasmids for each binding site in the 3’UTR of mRNA from potential target genes based on microRNA.org information. Then we confirmed miR-182-5p binding to the 3’UTR of target gene mRNA by luciferase assay with miR-182-5p precursor. PmirGLO Dual-Luciferase miRNA Target Expression Vector was used to perform 3’UTR luciferase assay (Promega, Madison, WI, USA). The primer sequences used for plasmid inserts are shown in **Table 2**. In a total volume of 20 µl, 5 µl each of 100 µM forward primer and reverse primer, 2 µl of 10x annealing buffer (100 mM Tris-HCl, pH 7.5, 1 M NaCl, 10 mM EDTA) and 8 µl water were added to a 200 µl PCR tube and incubated at 95 °C for 5 minutes then placed at room temperature for 1 hr. The oligonucleotides were ligated into the *Pme*I- *Xba*I site of pmirGLO Dual-Luciferase miRNA Target Expression Vector. Colony direct PCR was performed for insert recognition using REDTaq (Sigma, St. Louis, MO, USA). The primers used for PCR were as follows: forward primer, 5′-cgtgctggaacacggtaaaa-3′; reverse primer, 5′-gcagccaactcagcttcctt-3′. PCR parameters for cycling were as follows: 94°C for 3 minutes, 30 cycles of PCR at 94°C for 30 seconds, 55°C for 30 seconds and 72°C for 30 seconds, 72°C for 10 minutes and 4°C for 10 minutes. The PCR product was digested with NotI (TaKaRa/Fisher Scientific, Pittsburgh, PA, USA). The sizes of vectors containing inserts were about 200 bp and 100 bp by electrophoresis since the NotI recognition sequence was incorporated into the primers.

**Table 2:** Primer sequences used for plasmid construction.

name		sequence	
FOXF2- 1S	5'	AAACTAGCGGCCGCTAGTctacatttggaTACAGGTGCCAAaT	3'
FOXF2- 1AS	5'	CTAGAtTTGGCACCTGTAtccaaatgtagACTAGCGGCCGCTAGTTT	3'
FOXF2- 2S	5'	AAACTAGCGGCCGCTAGTtaTGTATACTTTTACAGTTTGCCAAaT	3'
FOXF2- 2AS	5'	CTAGAtTTGGCAAACTGTAAAAGTATACAtaACTAGCGGCCGCTAGTTT	3'
MTSS1-1S	5'	AAACTAGCGGCCGCTAGTctTTTGATTTTTCTGAAGGTGCCAAaT	3'
MTSS1- 1AS	5'	CTAGAtTTGGCACCTTCAGAAAAATCAAAagACTAGCGGCCGCTAGTTT	3'
MTSS1- 2S	5'	AAACTAGCGGCCGCTAGTctTGGAGTCTAGCAACTTGCCAAtT	3'
MTSS1- 2AS	5'	CTAGAaTTGGCAAGTTGCTAGACTCCAagACTAGCGGCCGCTAGTTT	3'
MTSS1- 3S	5'	AAACTAGCGGCCGCTAGTTTGAACACTGCTGCGATTGCTCAAGT	3'
MTSS1- 3AS	5'	CTAGACTTGAGCAATCGCAGCAGTGTTCAAACTAGCGGCCGCTAGTTT	3'
MTSS1- 4S	5'	AAACTAGCGGCCGCTAGTaaTCTGAATAAATAAAATGTTGCCAAaT	3'
MTSS1- 4AS	5'	CTAGAtTTGGCAACATTTTATTTATTCAGAttACTAGCGGCCGCTAGTTT	3'
RECK-S	5'	AAACTAGCGGCCGCTAGTgcTGCTACTTATATAATTGCCAAaT	3'
RECK-AS	5'	CTAGAtTTGGCAATTATATAAGTAGCAgcACTAGCGGCCGCTAGTTT	3'
FOXF2- MU-1S	5'	AAACTAGCGGCCGCTAGTctacatttggaCCGAGGCTGGCCaT	3'
FOXF2- MU-1AS	5'	CTAGAtGGCCAGCCTCGGtccaaatgtagACTAGCGGCCGCTAGTTT	3'
FOXF2- MU-2S	5'	AAACTAGCGGCCGCTAGTtaCTCATCCCCTCCGAGTCCTGGCCaT	3'
FOXF2- MU-2AS	5'	CTAGAtGGCCAGGACTCGGAGGGGATGAGtaACTAGCGGCCGCTAGTTT	3'
MTS1 MU-1S	5'	AAACTAGCGGCCGCTAGTctCTCTCTTTCCGCGAAGGCTGGCCaT	3'
MTS1 MU-1AS	5'	CTAGAtGGCCAGCCTTCGCGGAAAGAGAGagACTAGCGGCCGCTAGTTT	3'
MTS1 MU-2S	5'	AAACTAGCGGCCGCTAGTctCTTCTCGCCGGCACCCTGGCCtT	3'
MTS1 MU-2AS	5'	CTAGAaGGCCAGGGTGCCGGCGAGAAGagACTAGCGGCCGCTAGTTT	3'
MTS1 MU-3S	5'	AAACTAGCGGCCGCTAGTaaCCCTCACAAACAACATGCCTGGCCaT	3'
MTS1 MU-3AS	5'	CTAGAtGGCCAGGCATGTTGTTTGTGAGGGttACTAGCGGCCGCTAGTTT	3'
MTS1 MU-4S	5'	AAACTAGCGGCCGCTAGTttgaacCCCGGCGGGCCCTGTGCCgT	3'
MTS1 MU-4AS	5'	CTAGAcGGCACAGGGCCCGCCGGGgttcaaACTAGCGGCCGCTAGTTT	3'
RECK-MU-S	5'	AAACTAGCGGCCGCTAGTgcCTCTCCCCACCTACCCTGGCCaT	3'
RECK-MU-AS	5'	CTAGAtGGCCAGGGTAGGTGGGGAGAGgcACTAGCGGCCGCTAGTTT	3'
MTSS1 NheI cloning forward primer	5'	aaaGCTAGCcctcgccctgccggagccgggaaaATGgag	3'
MTSS1 XhoI cloning reverse primer	5'	aaaCTCGAGttgtgaacctaagaaaagcgaggggctgag	3'
RECK NheI cloning forward primer	5'	GCTAGCggccaagctgggtccgagcatcccg	3'
RECK XhoI cloning reverse primer	5'	CTCGAGcaactacaaaccagcagtcctgaat	3'

For miR-182-5p precursor transfection, prostate cancer cells were co-transfected with miR-NC and pmirGLO or miR-182-5p and pmirGLO Dual-Luciferase miRNA Target Expression Vectors using Lipofectamine 2000 (Invitrogen). Luciferase activity was assessed using the Dual-Luciferase® Reporter Assay System (Promega) (48 hours after transfection).

### Establishment of stable low miR-182-5p expressing prostate cancer cells and effect on *in vivo* tumor growth

In order to observe the *in vivo* effect of miR-182-5p inhibitor on prostate cancer cells, we established stable low miR-182-5p expressing prostate cancer cell lines based on the lenti-viral system. We then used an *in vivo* nude mouse xenograft model. Lentivirus transfection was performed using a Lenti-Pac HIV Expression Packaging Kit (catalog #; HPK-LvTR-20, GeneCopoeia, Rockville, MD, USA) according to the manufacturer's instructions. Namely hsa-miR-182-5p inhibitor vector (catalog #; HmiR-AN0239-AM03, GeneCopoeia, Rockville, MD) or a miRNA inhibitor scrambled control clone pEZX-AM03 (catalog #; CmiR-AN0001-AM03, GeneCopoeia, Rockville, MD) with Lenti-Pac HIV mix were transfected into 293 Ta cells (GeneCopoeia) and incubated for 14 hours at 37°. The medium was then replaced with fresh medium containing 1/500 volume of the TiterBoost reagent. After 48 hours of incubation at 37°, the supernatant containing lentiviral particles was centrifuged at 500 x g for 10 min, filtered using 0.45 µm PES filter (Whatman/Fisher Scientific) and collected to sterile tubes. PC-3 cells were infected with lentiviral particles using Polybrene (8 µg/ml) (Sigma-Aldrich). After 24 hours incubation at 37°, medium was replaced with fresh medium to exclude Polybrene. Stably transfected cells (miR-182-5p low expressing or control) were selected using Hygromycin (100 µg/ml, Invitrogen, Carlsbad, CA, USA) for three weeks. Stable low miR-182-5p expressing prostate cancer cells (1×10^7^/each) or control cells (1×10^7^/each) were injected subcutaneously into the right back side flanks of 5 week-old nu/nu mice, respectively. A total of 8 nude mice (4-control, 4-miR-182-5p inhibitor) (strain BALB/c nu/nu; Charles River Laboratories, Inc., Wilmington, MA) were used for the *in vivo* xenograft model. MiR-182-5p expression was confirmed by real-time PCR when stable cell lines were injected into mice and when xenograft tissues were harvested. Tumor size was determined with calipers twice per week for 6 weeks, and tumor volume was calculated on the basis of width (x) and length (y): x^2^y/2, where x<y.

### Overexpression of target genes (RECK, MTSS1) and functional analyses

In order to construct target gene (RECK, MTSS1) over expressing plasmids, the genes were amplified with total RNA from human adult normal kidney tissues (catalog#: R1234142-50, Biochain Institute, Newark, CA) and RWPE-1 by transcription–polymerase chain reaction (RT-PCR). The sequences of primers for cloning are shown in **Table 2**.

Polymerase chain reaction products were cloned into the pTargeT-Mammalian Expression Vector System (Promega, Madison, WI). Then pCMV6-RECK or pCMV6-MTSS1 was obtained by subcloning a NheI–XhoI fragment from pTargeT-RECK/MTSS1 into the NheI–XhoI site of pCMV6-Entry Vector. Initially we transfected pCMV6-empty and pCMV6-RECK or –MTSS1 into prostate cancer cells and RNA and protein were extracted. Overexpression of RECK or MTSS1 was confirmed by real time RT-PCR and Western Blot analysis and functional analyses were performed.

### Effect of FOXF2 knock down on prostate cancer cell lines

We were not able to successfully make a FOXF2 over-expressing plasmid. Thus we performed functional analysis of FOXF2 using si-FOXF2 RNA in prostate cancer cell lines (PC3 and DU145). PC cells were transfected with FOXF2 si-RNA (Silencer Select si-FOXF2; Ambion) or negative control si-RNA [si-negative control (si-NC); Ambion] according to the manufacturer’s instructions. Briefly, cells were grown in six-well plates and transfected individually with the siRNA at a concentration of 200 pmol/well. The siRNA sequences are as follows: si-FOXF2- Sense; 5’-GAAAAGAUUUCGUCCUCAAtt-3’, Anti-sense; 5’-UUGAGGACGAAAUCUUUUCtg-3’. Transfection was performed with X-tremeGene siRNA Transfection Reagent (Roche Diagnosis, Basel, Switzerland). MTS, *in vitro* transwell invasion and wound healing assays were performed for functional analyses. For wound healing assay, after setting the wound, wound closure was assessed after 8 hours.

### Statistical analysis

All statistical analyses were performed using StatView (version 5; SAS Institute Inc., NC). Error bars in all the figures represent S.D. (Standard Deviation). Statistical significance was determined using the Students t-test or Analysis of Variance (ANOVA) for functional analysis. Chi-square or the Fisher’s exact test was used for the association of clinical parameters with miR-182-5p expression. A *p*-value of <0.05 was regarded as statistically significant.
